# Prototype Development: Context-Driven Dynamic XML Ophthalmologic Data Capture Application

**DOI:** 10.2196/medinform.7465

**Published:** 2017-09-13

**Authors:** Peggy Peissig, Kelsey M Schwei, Christopher Kadolph, Joseph Finamore, Efrain Cancel, Catherine A McCarty, Asha Okorie, Kate L Thomas, Jennifer Allen Pacheco, Jyotishman Pathak, Stephen B Ellis, Joshua C Denny, Luke V Rasmussen, Gerard Tromp, Marc S Williams, Tamara R Vrabec, Murray H Brilliant

**Affiliations:** ^1^ Marshfield Clinic Research Institute Biomedical Informatics Research Center Marshfield, WI United States; ^2^ Marshfield Clinic Research Institute Center for Oral and Systemic Health Marshfield, WI United States; ^3^ Marshfield Clinic Department of Ophthalmology Marshfield, WI United States; ^4^ Essentia Institute of Rural Health Center for Research and Education Duluth, MN United States; ^5^ Center for Genetic Medicine Northwestern University Chicago, IL United States; ^6^ Weill Cornell Medical College Healthcare Policy and Research Cornell University New York, NY United States; ^7^ Personalized Medicine Institute Mount Sinai New York, NY United States; ^8^ School of Medicine Biomedical Informatics Vanderbilt University Nashville, TN United States; ^9^ Division of Health and Biomedical Informatics Feinberg School of Medicine Northwestern University Chicago, IL United States; ^10^ Autism and Developmental Medicine Institute (ADMI) Geisinger Danville, PA United States; ^11^ Genomic Medical Institute Geisinger Danville, PA United States; ^12^ Department of Ophthalmology Geisinger Danville, PA United States; ^13^ Marshfield Clinic Research Foundation Human Genetics Marshfield, WI United States

**Keywords:** electronic health records, ophthalmology, data acquisition, extensible markup language XML, data collection

## Abstract

**Background:**

The capture and integration of structured ophthalmologic data into electronic health records (EHRs) has historically been a challenge. However, the importance of this activity for patient care and research is critical.

**Objective:**

The purpose of this study was to develop a prototype of a context-driven dynamic extensible markup language (XML) ophthalmologic data capture application for research and clinical care that could be easily integrated into an EHR system.

**Methods:**

Stakeholders in the medical, research, and informatics fields were interviewed and surveyed to determine data and system requirements for ophthalmologic data capture. On the basis of these requirements, an ophthalmology data capture application was developed to collect and store discrete data elements with important graphical information.

**Results:**

The context-driven data entry application supports several features, including ink-over drawing capability for documenting eye abnormalities, context-based Web controls that guide data entry based on preestablished dependencies, and an adaptable database or XML schema that stores Web form specifications and allows for immediate changes in form layout or content. The application utilizes Web services to enable data integration with a variety of EHRs for retrieval and storage of patient data.

**Conclusions:**

This paper describes the development process used to create a context-driven dynamic XML data capture application for optometry and ophthalmology. The list of ophthalmologic data elements identified as important for care and research can be used as a baseline list for future ophthalmologic data collection activities.

## Introduction

### Background

Capturing clinical information in a machine-interpretable format is challenging yet extremely critical to patient care, biomedical research, health care quality, and workflow efficiency initiatives [1-7]. However, without structured data capture, computers cannot be used to perform or enhance many tasks surrounding a patient encounter (eg, appointing, diagnostic test ordering, return of test results with interpretation, and billing) or to present treatment options to clinical staff [8,9]. In addition, some research, quality assessment, and process improvement activities may be cost prohibitive because of manual information review.

Electronic health records (EHRs) use a variety of mechanisms to capture structured (or coded) medical information to meet high-volume regulatory and billing requirements, but they often lack options for lower volume ancillary or practice-specific medical initiatives or research. As a result, health care institutions often supplement EHR data capture functionality with vendor software solutions, form development tools, or standard office tools (eg, spreadsheets and document editors) to create such capability [10-12]. These solutions usually have EHR integration limitations, are platform or device dependent, are difficult to maintain, lack security, and are limited in the ability to capture and use a broad range of clinical data capture methods. Not all of these solutions have exposed application program interfaces (APIs) that can be used to interface with EHRs, and there are challenges of maintaining functionality with the upgradation of the form system or EHR. Additionally, there are Web-based subscriptions or paid services that provide varying implementations of Web forms, but these have limited feature sets resulting in the inability to create custom questions, are unable to support system integration and illustration functionality requirements, and lack regulatory assurances needed for handling medical data (eg, Health Insurance Portability & Accountability Act [HIPAA] Security; [13-15]).

Processing information found within a document can be accomplished using a flexible machine and human interpretable markup language referred to as extensible markup language (XML). XML can be used to create Web-based data capture forms supporting a variety of common data formats, including text, numeric, coded results (eg, multiple choice), voice clips, and images. There are a number of Web-based data capture applications reported in the literature that have been used for research [16-19] and clinical care [20,21]. Notable innovations such as the dynamic generation of structured data entry forms using metadata stored in databases [20] or XML schemas, XML schema designer tools [18,22], or the integration of EHR data into an XML form [17] are reported and could be used.

Optometry and ophthalmology are specialty areas in health care that deal with the anatomy, function, and diseases of the eye and its surrounding structures. These specialties have been slow to adopt EHR or electronic data capture technology because of characteristically high-volume practices with complex workflows (eg, office visit to surgical suite transitions) and image-intensive documentation requirements [23-26]. Optical Coherence Tomography (OCT) specialty testing images, for example, can be captured by an external camera and scanned into the EHR as a PDF, thereby trapping numeric data in images as opposed to discretely computable fields. Much of the clinically relevant information is captured with hand-drawn illustrations of the eye, thus making it unavailable for subsequent clinical or research use unless manually interpreted [27]. In some countries, procedure reimbursement of nonphotographic retinal images relies on examiner-generated illustrations [28], and as many electronic drawing tools are not yet sophisticated enough to execute retinal illustrations, they reinforce the use of paper in clinical practice.

### Objective

Finding or developing software solutions that can easily integrate with and supplement basic EHR data capture functionality for complicated workflows are necessary for patient care, research, and quality management efforts [29]. Using the ophthalmologic areas as a use case for prototype development, we describe the process for identifying clinical- and research-relevant data elements and the creation of an open-source, context-driven, ophthalmologic data capture application that can be easily adapted to a variety of clinical workflows and EHR environments. This work takes advantage of previous research efforts [20] that use metadata encapsulated in a database or an XML schema to drive form generation and context-driven controls to populate the data capture application. We expand on these approaches by applying the technologies in an integrated fashion to the ophthalmologic areas and add ink-over Web controls to capture unstructured clinically relevant drawings and notes. Ink-over, often referred to as digital ink, refers to technology that digitally represents handwritten notes and drawings. In this study, we describe the process used to develop an ophthalmologic data capture prototype application and its heuristic evaluation.

## Methods

### Goal

The overall goal of this research was to develop a context-driven dynamic XML ophthalmologic prototype that enables efficient data capture of ophthalmologic and optometric data and increases the collection of discrete (structured) data while preserving the ability to capture handwritten notes and drawings where needed.

The specific objectives included: (1) determining clinical- and research-relevant ophthalmologic data elements (both structured and image-based); (2) documenting system requirements for an ancillary data capture application; and (3) designing, developing, and evaluating a data capture prototype that is context-aware, modifiable, and can integrate into an EHR.

### Environment

Marshfield Clinic Health System’s CattailsMD EHR has been used for clinical care since the late 1980s, serving clinicians throughout Central and Northern Wisconsin. It uses a variety of data-gathering techniques to capture and code patient encounter information, including diagnoses, laboratory results, procedures, medications, and vital sign measurements such as height, weight, and blood pressure. Clinical narratives and illustrations are stored in textual and/or image-based unstructured formats and made available for viewing via the EHR. Medical staff use tablet personal computers (PCs) to interact with EHR applications that have been optimized to run on these devices. Ophthalmology and Optometry departments use these EHR applications, but they also supplement clinical data capture by using paper-based forms that are scanned into the EHR.

### Process

**Figure 1:**
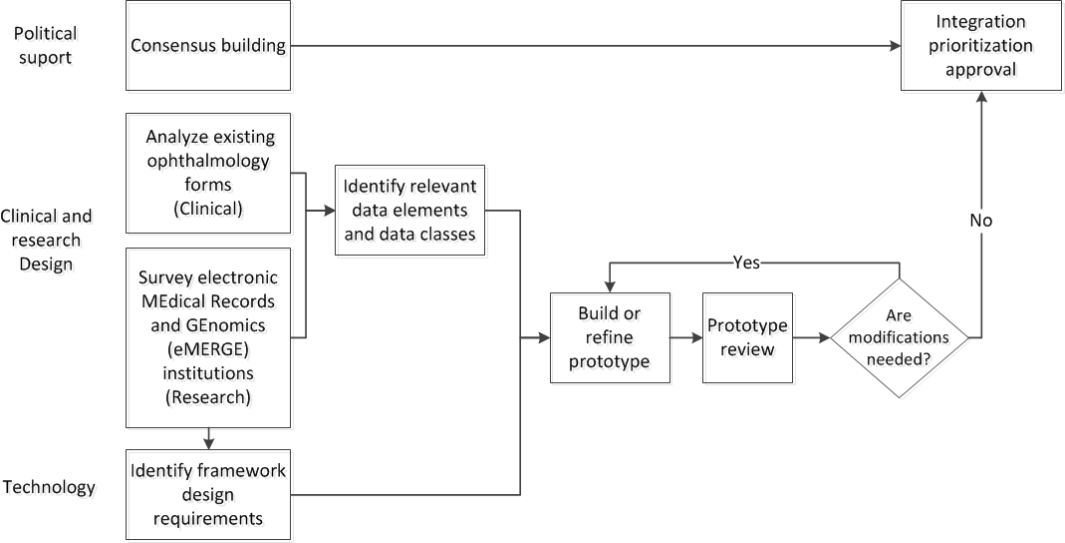
Process for developing the ophthalmologic data capture prototype.

We used a participatory process to develop the ophthalmologic data capture prototype. A design team comprising stakeholders (3 physicians, 2 administrators, 3 medical assistants, 2 researchers, 2 informaticians, and 2 programmers) conducted a series of face-to-face meetings to (1) gather and understand user and technology requirements and workflows within ophthalmology and optometry and (2) define and analyze existing and proposed data elements for capture. The latter prompted content analysis of existing forms and a survey. Both of these activities are described in the following section. Requirements identified from the design team discussions were developed into use cases for prototype development. For example, the physicians identified the need to capture drawings of the eye within the prototype. An overview of the prototype development process is shown in Figure 1. An iterative clinical review process was used to refine the prototype. The developed prototype was then reviewed and considered for integration into the EHR via a prioritization process.

### Clinical and Research Requirements

We conducted content analysis on 30 different handwritten unstructured paper forms used for documenting patient encounters within optometry and ophthalmology. Data elements nominated for prototype inclusion were prioritized in the following order: (1) data elements found on multiple forms or used by multiple practices and/or specialists; (2) data elements required for Meaningful Use [30]; and (3) data elements found on the Comprehensive Adult Medical Eye Evaluation developed by the American Academy of Ophthalmology (AAO) [25]. This list was then reviewed and prioritized for inclusion by the design team. In addition, each data element was defined and then grouped into a logical data class (ie, visit information, medical history, family history, examination, slit-lamp examination, specialty testing, and miscellaneous).

In a parallel effort, we distributed an EHR ophthalmologic data availability survey to institutions participating in the electronic MEdical Record and GEnomics (eMERGE) network [31] to gain an understanding of data elements that other institutions capture and to evaluate the generalizability and common types of ophthalmologic data elements collected across institutions and the mechanisms used for their capture. The survey can be found in Multimedia Appendix 1.

### Technology and Architecture

**Figure 2:**
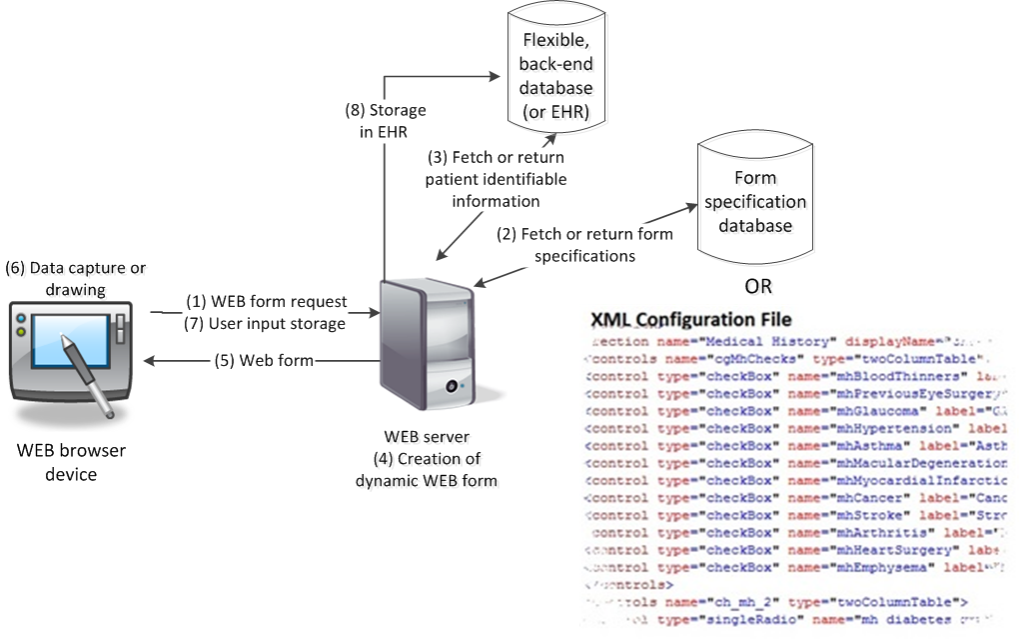
Context-driven dynamic extensible markup language (XML) architecture.

A prototype of the ophthalmologic data capture application was developed on an extensible, open architecture using Microsoft’s ASP.NET MVC 3.0, JavaScript, and jQuery. The development environment and database chosen for prototype development was based on the familiarity and experience of the programming team. An overview of the architecture is shown in Figure 2, and the database schema to support the data capture application can be found in Multimedia Appendix 2. This architecture supports a *form specification database* (FSD), which includes form design specifications, actions, and Web control definitions. Form specifications can also be stored in XML configuration files if a database is not available or for lightweight implementations. Each data collection form is constructed using Web controls (hereafter referred to as a *control*), which allows a user to interact with the Web form *.* The FSD has many control records in the database to define a form both in appearance and actions. The FSD schema can store multiple forms and is extensible to accommodate additional control types that may be added at a later date.

The overall process for rendering a Web form is outlined in Figure 2 and is described below:

- A Web server generates the Web form based on specifications stored in the FSD and brokers data requests between the *EHR* or *flexible back-end database* (FBD) and the Web browser device (steps 1, 2, and 4).
- The Web server then requests patient identifiable information (PII) from the EHR or FBD to fill the Web form (step 3).
- The Web server generates the Web form, includes the PII, and presents it to the user (steps 4 and 5).
- User data and ink-over annotated drawings are captured and passed back to the EHR or FBD for storage (steps 6-8).

A collection of control classes are used to parse the XML input (retrieved from either the FSD or an XML configuration file) and then deliver the form content to the user via the Web browser. A control can be interactive (a form element that the user can act upon—CheckBox, TextBox, etc) or passive (eg, a container for grouping controls in a visual manner). Each control stored in the FSD contains a number of properties and methods as described in Table 1. These properties and methods define how the form will display and act when presented to the user. The Web form can dynamically change by modifying the controls and/or control properties and methods defined in the FSD or XML configuration file.

**Table 1:** Web control properties and methods.

Type	Web control class	Description
**Properties**		
	Name of control	A unique identifier for the specific element on the form. Multiple controls of the same type will have different names.
	Label	Human readable text to display to the user (not required).
	Control type	The type of the control (CheckBox, TextBox, ComboBox, image, etc). Once a <control/> element is found in the extensible markup language (XML), type property is checked to determine the control class to use.
	Value	The current value of the property the control represents. This value can be preloaded from the database or assigned as a result of the form submission.
	Children	The collection of child controls whose visibility is dependent on the value of the current control. For example, an ink-over image control can be displayed if a CheckBox is checked.
	Functions	The collection of events that define the logic for displaying or hiding child controls. This can be further expanded to handle non–child-related events (notifying the user, requesting more information from the server, etc).
**Methods**		
	Render hypertext markup language (HTML)	Each control is responsible for rendering itself. A CheckBox type control only knows how to render a CheckBox. This is also applicable for a TextBox, ComboBox, or any other basic or complex control type in the library. This means that a control is ignorant of its parent and sibling controls (if any) and only cares *where* its children are rendered, not *how*. This allows flexibility of form layout and control hierarchy.
	Render JavaScript	Whereas the HTML and JavaScript work hand-in-hand, the two are not usually rendered at the same place on the HTML page, so the two must be separated to meet the needs of a Web-based application.
	Render XML	After a user provides results and submits the form, the results are rendered back into XML format. That XML can then be sent to a service/database for storage or simply saved as a file on the local system. Similar to the Render HTML method details state, each control is responsible for how it’s rendered in the resulting XML, whereas a CheckBox control’s value may only be *checked* or *unchecked*; some other complex control will have a collection of results.

**Figure 3:**
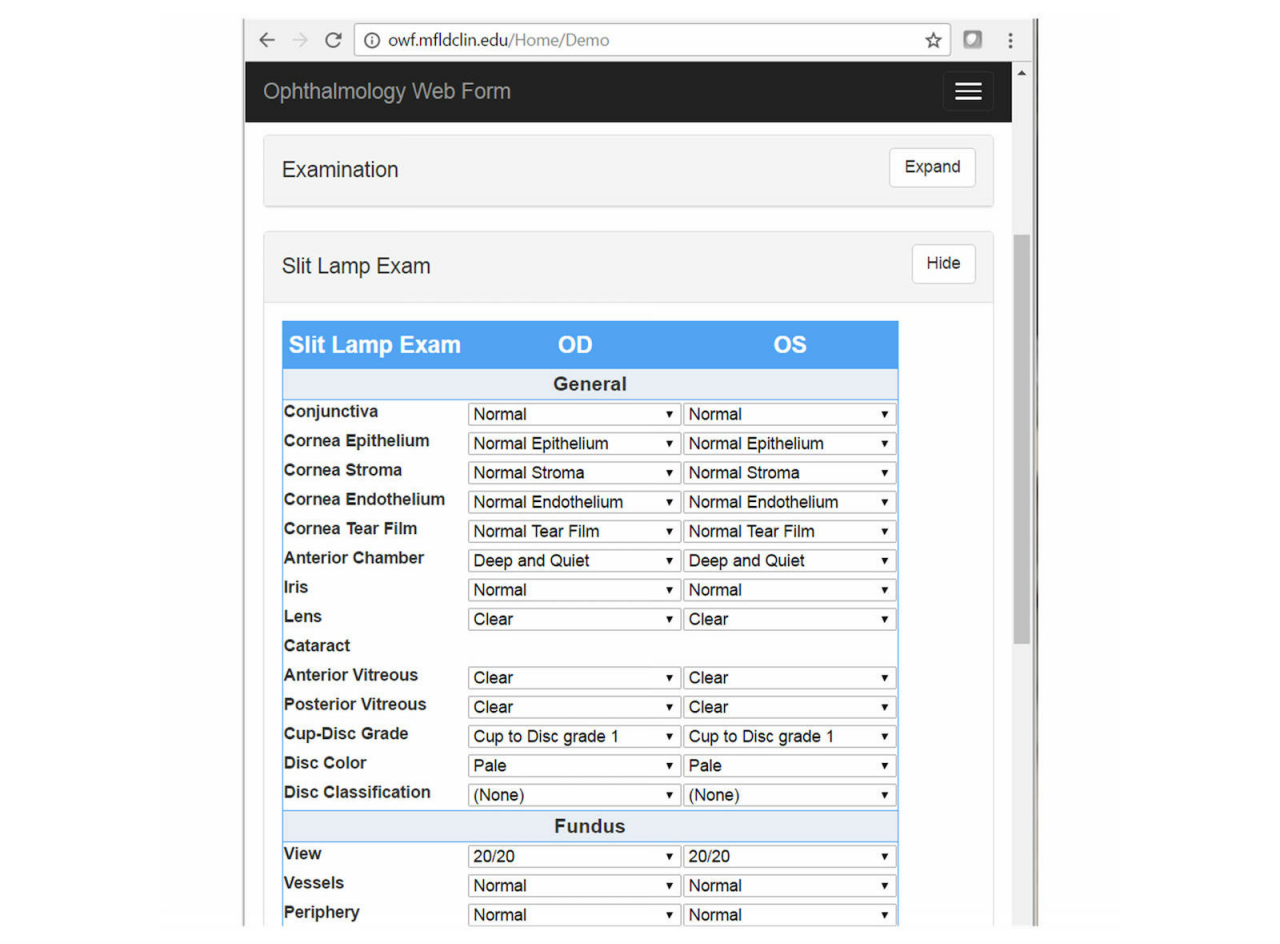
Ophthalmology prototype with expanded slit-lamp exam section.

The ophthalmology data capture prototype is Web enabled to run on a variety of devices, including an iPad. The form is divided into seven logical sections for data entry. All the sections are initially collapsed for easy form navigation and then expanded as data collection ensues. Figure 3 shows the Web form with the section detail hidden, with the exception of the slit-lamp examination section.

#### Context-Driven Controls

**Figure 4:**
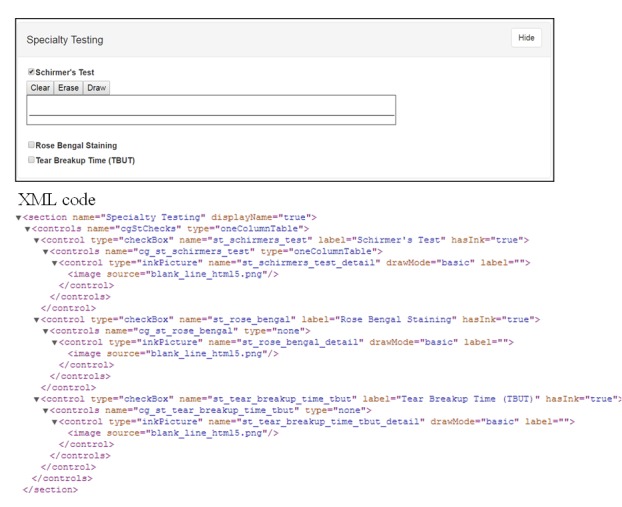
Specialty control and corresponding extensible markup language (XML) code.

**Figure 5:**
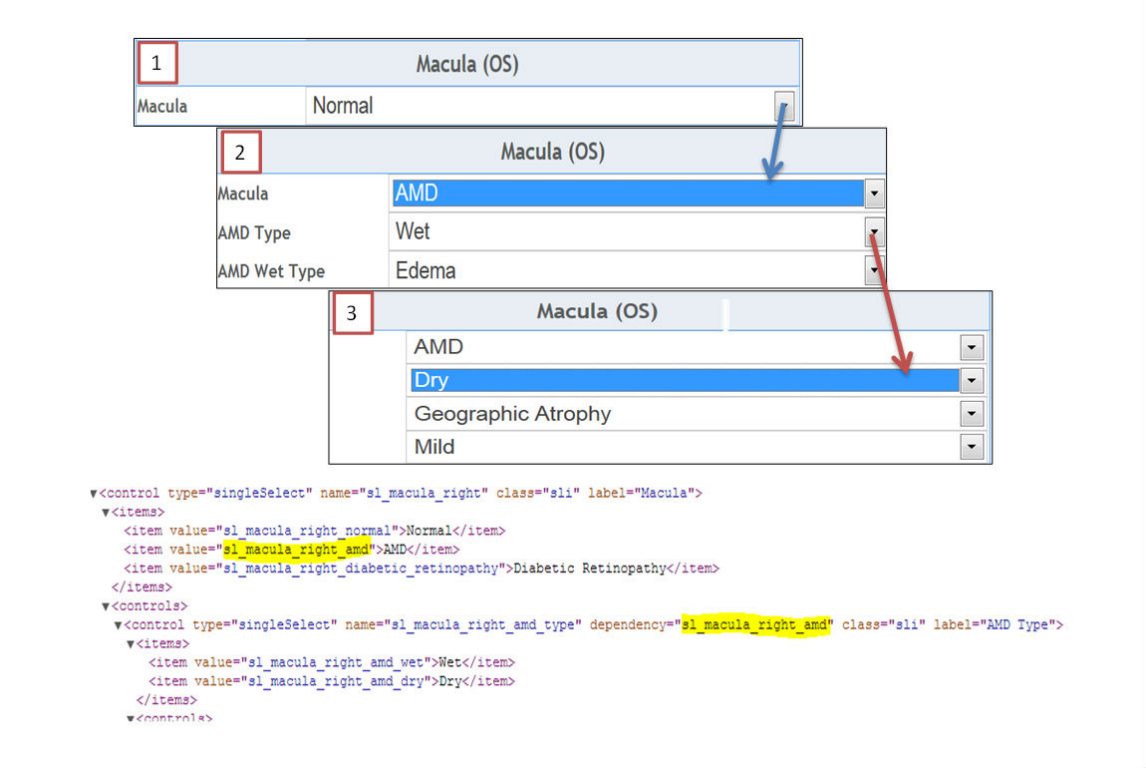
Example for adding the show/hide event to a dependent control and corresponding extensible markup language (XML) code.

The inclusion of context-driven controls is considered an important component of the application for several reasons. First, it reduces the need for a user to review and answer unnecessary questions. This helps to both guide and support a streamlined workflow for data entry. Figure 4 shows one section of the ophthalmology prototype that supports hidden controls. When a clinician selects a specialty test, a control is displayed and readied for handwritten comments. The corresponding XML used to generate the form is pictured in Figure 4. Second, it helps to keep the data entry form as compact as possible, removing unnecessary questions from the form, based on the answers to prior questions. For example, in Figure 5, we have a control for describing the macula or central retina. If the patient has a normal macula, Figure 5 (1) is only shown with no additional data entry required. The selection of a drop-down value for *Macula* (2) causes another form to appear, prompting the user to define the type of macular disorder, which, in this example, has been shown as age-related macular degeneration (AMD). The selection of AMD prompts additional controls (3) based on the attribute selected for *AMD Type*, thus resulting in additional relevant data capture. Currently, the application allows for very basic context-driven behavior (showing/hiding child controls based on a selected value). This is done by making use of a dependency property associated with the control and using naming conventions within the Form Specification Database (Figure 2). Figure 5 also shows the corresponding XML code that demonstrates this concept. To display a control when a value from a drop-down list is selected, the name of that control needs to match the value of the item on which it is dependent. The XML code is automatically generated based upon the records found in the Form Specification Database (Figure 2). Users are able to customize and/or add new form features by either adding or removing records or changing the attributes of a data element within the database.

#### Ink-Over Capability

**Figure 6:**
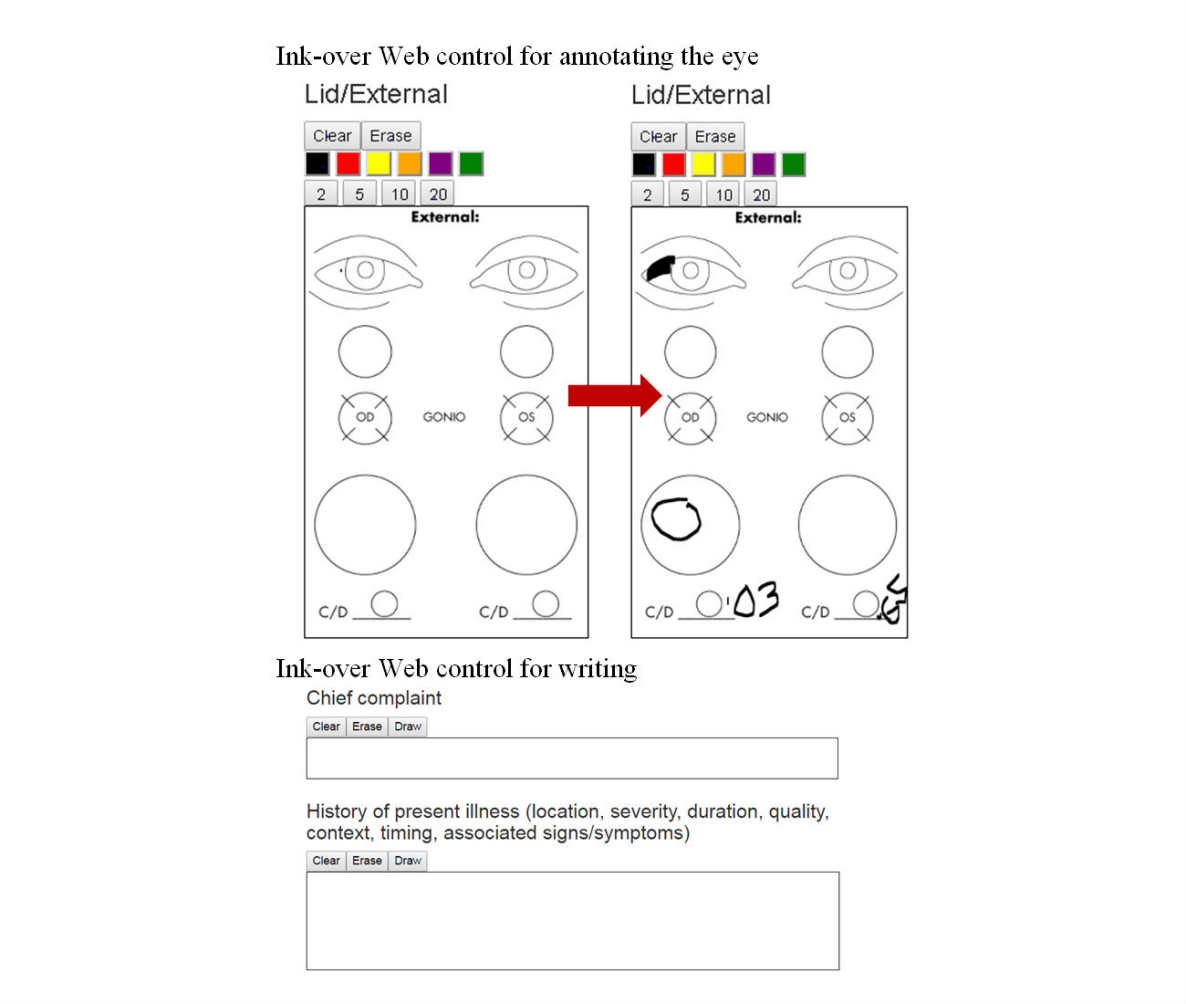
Examples of ink-over controls.

Ophthalmologists require tools to illustrate ocular abnormalities [28]. A key feature of this application is the ability to draw on a Web form or canvas. The canvas area is overlaid with a transparent image, and the user can annotate as they see fit on the transparent image as shown in Figure 6. The images can be specific to the context of the form, as in the ink-over Web control for annotating the eye or simply a series of horizontal lines for writing text annotations (Figure 6). Given Marshfield Clinic Health System’s information technology environment (Tablet PCs and Windows 7/Internet Explorer [IE] 8), we focused on embedding the Microsoft.Ink library into the Web form to provide native ink-over functionality to the users. For users accessing the form on a device with browsers such as IE 9+, Chrome, Firefox, Safari, and others that do not have the Microsoft.Ink libraries installed, HTML5 Canvas was used. The resultant images are saved along with the coded data to the EHR or FBD (Figure 2).

### Prototype Evaluation

We conducted a heuristic evaluation of the software following the development of the prototype. Heuristic evaluation is a usability inspection method employed to discover issues with a user interface. A usability analyst at the Marshfield Clinic Research Institute reviewed the ophthalmology data capture prototype multiple times, comparing it with a list of established heuristics, recording and reporting all conflicts with the heuristics [32].

## Results

The most important design criteria prioritized by clinical, research, and technical stakeholders during the interview and survey processes are presented in Table 2. Ophthalmologists considered the most important requirements for an interface, which included an interface that is (1) easy to navigate, (2) integrated into clinical workflow, (3) organized into a consolidated view and meaningful groups (meaning all ophthalmologic information is located in one place so clinicians can view it easily, and groupings represent visit reason, medical and family history, examination, and specialty testing), (4) EHR integrated to prefill the forms with relevant patient information, and (5) ink-over form writing capable to support ocular illustrations. The important technical requirements focused on integration and maintenance functionality. Research requirements highlighted coded data capture and sharing of the prototype technology with other collaborators. All stakeholders mentioned the need for a secure data sharing and authentication architecture. Our solution addresses all of these requirements.

**Table 2:** Stakeholder requests that influenced prototype design.

Item	Stakeholder	Description
Consolidated form view	Clinical	Consolidate the 30 forms into a single form, with logical groupings for medical and family history, visit information, examination, and slit-lamp examinations. Collect data once and display in multiple views.
Electronic health record (EHR) integration	Clinical	Prefill form with EHR data, including patient identifiers and patient and family history. Data captured in the form should be sent to the clinical data repository of the EHR for use in patient care and research.
Multiple data capture formats	Clinical and Research	System supports Boolean (true/false), numeric, coded, textual, and analog (graphical) data inputs. Allows for flexibility of data capture while still maintaining the ability to capture discrete and disparate data types.
Context-driven controls	Clinical	Behavior of a form control can be determined by the values of another control. Facilitates conditional questions to be enabled, as necessary, based on the answers to other questions and gives the form a neat and organized appearance.
Metadata driven	Technical	Form specifications should be easily amendable to accommodate new or modified data capture requirements and form layout changes among practices. Properties of the dynamic Web controls can be easily modified in the database, thus allowing changes to the extensible markup language (XML) specifications.
Dynamic form generation	Technical	Dynamic generation of the form, based on a form definition. Storage of multiple types or versions of forms.
Flexible, extensible back-end database	Technical	Back-end database will support storage and retrieval of multiple data types, allowing for capture and storage of discrete, disparate data types.
Service-oriented architecture (SOA) and database agnostic	Technical	Utilize SOA. The SOA layer will allow for enhanced security and consistency in data transfers.
System portability	Research	Architecture used for this development can either be a stand-alone system or have the ability to integrate into an EHR.
Open source	Research	Architecture should be shareable with other research sites that do not have access to ophthalmologic data collection mechanisms.
Secure data sharing/Authentication	All	Architecture should support integrated user login and secure data sharing transparency between the application and EHR.

### Content Analysis

Unique data elements, identified from over 30 different clinical ophthalmology forms, were reviewed by clinicians and specialty leadership. Several collaborative discussions were held to organize the data elements into seven general data classes to support common ophthalmologic workflows as shown in Table 3. Ten data elements important to research were identified within these data classes (indicated with superscript letter *a* within Table 3). Medical and family history related data elements will likely require EHR access to display previously collected information. An API to the EHR can be used to collect extant data to prefill the form.

### eMERGE Data Availability Survey

Out of 9 eMERGE sites, 6 sites (66%; Geisinger Health System, Marshfield Clinic, Mayo Clinic, Mount Sinai, Northwestern University, and Vanderbilt University) completed the *Ophthalmology/Optometry Data Availability in the Electronic Health Record* survey found in Multimedia Appendix 1. Out of these 6 eMERGE sites, 4 sites (66%) used commercially developed EHRs (Epic and General Electric’s Centricity), and 2 sites used in-house developed EHRs (Vanderbilt and Marshfield’s CattailsMD). A summary of the survey responses can be viewed in Table 4. The ophthalmologic information from the respondents’ EHRs is captured in a variety of data formats, including coded, XML, text, and images. Many of the data elements identified by Marshfield are also being collected at other eMERGE institutions. As expected, there is only a limited amount of coded ophthalmologic data captured in the EHR among the eMERGE institution respondents.

**Table 3:** Data class groupings and data elements important to ophthalmologic health care and research.

Data class	Types of data elements		
Visit information	Chief complaint	History of present illness	
Medical history	Blood thinners	Asthma	Diabetes
	Previous eye surgery	Cancer	Smoking
	Macular degeneration	Stroke	Alcohol
	Glaucoma	Arthritis	Mental status
	Hypertension	Heart surgery	Occupation
	Myocardial infarction	Emphysema	
Family history	Blindness	Glaucoma	Macular degeneration
Examination	Visual acuity^a^	Medial rectus; oculus dexter (OD or right eye)	Intraocular pressure^a^
	Pinhole	Medial rectus; oculus sinister (OS or left eye)	Visual field defect^a^
	Current glasses	Lid/External	Pupils
	Current contacts		
Slit-lamp exam general	Conjunctiva	Anterior chamber	Posterior vitreous
	Corneal epithelium	Iris	Cup-disc ratio/grade^a^
	Corneal stroma	Lens-cataract grade and type^a^	Disc color
	Corneal endothelium	Anterior vitreous	Disc classification
	Corneal tear film		
Slit-lamp exam fundus	View	Vessels	Periphery
Slit-lamp exam macula	Normal	Diabetic retinopathy^a^	Age-related macular degeneration includes presence and type of drusen^a^
Specialty testing	Schirmer test^a^	Rose Bengal staining^a^	Tear breakup time^a^ (TBUT)
Miscellaneous	Impression	Return to clinic	Additional workflow coordination notes
	Recommendation		

^a^indicates that the data element is deemed important for research activities by research stakeholders.

### Prototype Evaluation

A heuristic evaluation of the prototype revealed that the form most often displayed inconsistent use of controls. When given the choice of the same number and types of options, the form contained both radio buttons and drop-down controls. For consistency and efficiency of use, the usability analyst recommended implementing radio buttons in all cases so that direct selection with a stylus was possible. The analyst also recommended that all text boxes have an erase toggle, rather than the existing erase button so that users can go back and forth between writing and erasing. The form originally prevented users from continued writing in text boxes after clicking the erase button. It required that the text box be cleared before accepting input. Minor recommendations included highlighting selected functionality, increasing padding around buttons and controls (radio buttons, drop-down boxes, text boxes, etc), and indenting dependent child controls to show relation to their parent control.

**Table 4:** The electronic MEdical Record and GEnomics (eMERGE) institution responses to ophthalmologic data availability in their electronic health records (n=6).

Data elements captured for right/left eyes	EHRs that capture data element, %	EHRs that capture coded/extensible markup language (XML) data elements, %	Data capture formats
Visual acuity	83	50	Coded or XML, image, text
Intraocular pressure	66	33	Coded or XML, image, text
Fundus exam	66	33	Coded or XML, image, text
Visual field exam	66	16	Image, text
Optical coherence tomography (OCT)	66	16	XML, image, text
Cup-disc ratio	83	50	Coded or XML, image, text
Presence of drusen	83	33	Coded, image, text
Soft drusen	83	33	Coded, image, text
Hard drusen	83	33	Coded, image, text
AMD (age-related macular degeneration) staging severity	50	16	Image, text
Severity of diabetic retinopathy	66	33	XML, image, text
Macular edema	83	16	Coded or XML, image, text
Severity of cataract	33	0	Image, text
Brightness acuity	50	16	Coded, image, text
Schirmer test (value)	50	16	Coded, image, text
Rose Bengal staining	50	0	Image, text
Tear breakup time (BUT)	33	0	Image, text
BUT measurement method	33	0	Image, text

## Discussion

### Principal Findings

This research describes the creation of a context-driven, dynamic XML data capture prototype for ophthalmological care and research. Its open architecture allows the use of a service-oriented architecture (SOA), which can facilitate integration with a variety of EHRs for retrieval of patient-specific information and the transfer of newly collected information back to the EHR, thereby making the data available for other uses. The architecture also supports Web-based forms that can be created dynamically from a database or an XML schema, context-based controls for efficient data entry, and ink-over forms for illustrating abnormalities of the eye. The source code, along with a demonstration version of the ophthalmology prototype, can be found on the Marshfield Clinic Research Institute’s website [33].

### Comparison With Prior Work

We investigated several well-known data capture solutions to use for ophthalmology prototype development [10-15,19]. Many of the solutions had limitations with inbound and/or outbound EHR data flows, supporting conditional logic, ink-over drawing capability, and licensing for operational use [19]. With the unique challenges presented in ophthalmology, we chose Web-based technology for prototype development because it minimized problems when running the application on multiple types of devices. The choice of this technology also provided the ability to share the prototype with other institutions by leveraging SOA for EHR integration.

We encountered several challenges. First, ophthalmologic workflows are complicated, as indicated by the current use of over 30 different paper-based forms. Creating a single, easy-to-navigate, Web-based user interface required analyzing over 140 unique data elements and employing several previously described methods to reduce the number of items and to determine logical groupings of data classes for efficient workflow. Form design was problematic because of the large number of data elements required for data capture. We developed a series of context-based controls to hide data capture complexity.

Second, the XML architecture used to support context-driven controls must be adaptable to changing data collection needs and new controls. To address this, we added attributes to each control within the database, indicating whether a control was dependent on another and the action needed to invoke the control. Using this approach enables one to easily modify form actions by changing database control configurations.

Third, providing Web-enabled drawing capability for eye abnormalities introduced some additional challenges. Our design required provisions for native ink-over drawing functionality in a large variety of Web-enabled devices, supporting a wide range of client configurations. We utilized Microsoft.Ink library for devices within Marshfield Clinic Health System’s computing environment but had to investigate other options for non-Marshfield devices with other operating system or browser requirements. We designed the ink-over capability to be device agnostic (functional on tablets, laptops, desktops, and mobile phones) as long as the administrative user defines a form with the appropriate ink-over controls. A significant effort was expended to support browser version detection and Microsoft.Ink compatibility.

The ophthalmology data capture prototype is currently packaged as a stand-alone application for demonstration purposes. We envision that the prototype will be packaged as an EHR add-on for use in ophthalmology and optometry departments. The prototype is built on SOA, and the architecture promotes context awareness and supports the transition of data between the application and EHR in a secure manner. This prototype currently uses a flexible back-end database—MySQL (Figure 2). This stand-alone database could easily be transitioned into an EHR data repository with the development of a wrapping Web service to broker EHR data exchange to and from the services of this prototype system. This level of integration would allow patient information from the EHR to be prefilled in the application’s forms and minimize data entry for the user.

### Limitations

The data capture application was reviewed by several ophthalmologists throughout the development process, and suggestions were provided for terminology and logical groupings of data elements and form flow. During the prototype development, we did not conduct a formal usability evaluation, but we did conduct a heuristic evaluation. Heuristic evaluations are usually hampered by the fact that reviewers who conduct them are not experts in the field that a user interface covers. In this case, a subject-matter expert was involved throughout the data gathering and design phases of development, so this evaluation may be less limited than others because of this previously contributed expertise. The modular nature of our application architecture permitted us to address the findings of the heuristic evaluation quickly. We were able to modify the type of controls used to provide consistent controls, and the enhancement to the text entry control was instantly applied across all forms using it.

Data elements collected using this application can be defined and annotated using standards set forth by the AAO, Clinical Data Interchange Standards Consortium (CDISC), or health care data standards consortium. Future application enhancements could include APIs that interface with common terminology databases or management systems.

Finally, we did not implement audit trails or identification management or explore the use of data entry error–checking algorithms within this prototype, as it was a proof-of-concept project and meant to explore the possibilities of dynamic form generation and context awareness for data collection. Future development will include this functionality.

### Conclusions

This research describes the creation of an open-source, context-driven, structured data capture dynamic XML ophthalmologic data capture application that can be integrated into a variety of EHRs. Relevant ophthalmologic and optometric data elements were identified for clinical care and research. Data entry was streamlined using context-driven controls and ink-over capabilities for illustrating eye abnormalities.

## Appendix

Multimedia Appendix 1 eMERGE Survey - Ophthalmology/Optometry data availability in the electronic medical record.

Multimedia Appendix 2 Database schema used to support the extensible markup language (XML)-driven data capture framework.

## Abbreviations

AAO: American Academy of Ophthalmology
AMD: age-related macular degeneration
API: application program interface
CDISC: Clinical Data Interchange Standards Consortium
CDXA: context-driven dynamic XML architecture
CTSA: Clinical and Translational Science Award
EHRs: electronic health records
eMERGE: electronic MEdical Record and Genomics
FBD: flexible back-end database
FSD: form specification relational database HIPAA: Health Insurance Portability & Accountability Act
HTML: hypertext markup language
NCATS: National Center for Advancing Translational Sciences
NHGRI: National Human Genome Research Institute
NIH: National Institutes of Health
OCT: optical coherence tomography
PCs: personal computers
PII: patient identifiable information
SOA: service-oriented architecture
TBUT: tear breakup time
XML: extensible markup language
