# Supplementary material for: Prototype Development: Context-Driven Dynamic XML Ophthalmologic Data Capture Application
Source: JMIR Med Inform. 2017 Sep 13;5(3):e27. doi: 10.2196/medinform.7465 (PMC5617903; doi:10.2196/medinform.7465)
Supplement: Multimedia Appendix 1 [file medinform_v5i3e27_app1.pdf]

eMERGE Survey - Ophthalmology/Optometry Data Availability in the Electronic Medical Record

1. What electronic health record does your organization use for Ophthalmology/Optometry (check all that apply)?
   a. _____ EPIC Version: ______________
   b. _____ General Electric
   c. _____ Cerner
   d. _____ Other:__________________________

2. Is ophthalmology/optometry data stored in the electronic health record?
   Yes _____ or No _____ (if no… you are done with this survey)

3. If yes, what is the format of the information stored (complete the table below using the following codes).
   C= coded or structured
   T= Text documents
   I = Image documents
   X=XML
   O=Other: ______________________

Priority		Data Element	Right Eye	Left Eye	Present but cannot differentiate eye
1	Glaucoma	Intraocular pressure (IOP)			
		Cup disk ratio			
		Visual field defect – Humphrey visual field analyzer data capture			
		Visual acuity			
	AMD	Fundus exam			
		Severity of AMD (Staging)			
		Presence of Drusen			
		- Soft drusen			
		- Hard drusen			
		Optical coherence tomography (OCT)			
	Diabetic Retinopathy (DR)	Fundus exam	a	a	a
		Severity of DR			
		Macular edema			
		OCT	a	a	a
		Visual acuity	a	a	a
Priority 2	Cataract	Severity			
		Visual acuity	a	a	a
		BAT Brightness Test			
	Dry eye	Shirmer's test result (value)			
		Rose Bengal staining			
		Tear breakup time (BUT)			
		BUT measurement method			

[a]There is some repetition of data elements. For easier understanding, the [a] data element was included under multiple conditions to present a comprehensive view of data elements for a disease/abnormality. Only complete the information for 1 of the data elements.
[a]Indicate C=coded or structured data; T=text documents; I = image documents; X = XML format/documents or O = other format (indicate the format).

Description/definition of the ophthalmology/optometry terminology.

AMD Staging – indication of the severity of AMD (http://www.aoa.org/documents/CPG-6.pdf).

BAT Brightness Test – is used to test glare disability.

Cataract severity – is measured in terms of numbers to indicate the severity of the cataract condition. The higher the number the more severe the cataract.

Cup-to-disc ratio – is a measurement used in ophthalmology and optometry to assess the progression of glaucoma. Normal subjects have a cup-disk-ratio around .2 to .3.

Diabetic Retinopathy Severity – a classification of severity or progression of the disease. (http://archive.icoph.org/pdf/Diabetic-Retinopathy-Detail.pdf)

Dilated fundus examination (DFE) – is a diagnostic procedure that employs the use of mydriatic eye drops (such as tropicamide) to dialate or enlarge the pupil in order to obtain a better view of the fundus of the eye. Once the pupil is dilated, examiners often use specialized equipment such as an ophthalmoscope or fundus camera to view the inner surfaces of the eye.

Drusen presence – recording of the number of soft drussen present when evaluating the eye.

Intraocular pressure (IOP) – is the fluid pressure inside the eye. Tonometry is a method used to determine IOP. Current consensus among ophthalmologists and optometrists define normal intraocular pressure as that between 10 mmHg and 20 mmHg.

Optical coherence tomography (OCT) – is an interferometric technique, typically employing near-infrared light. The use of relatively long wavelength light allows it to penetrate into the scattering medium.

Macular edema – occurs when fluid and protein deposits collect on or under the macular of the eye (a yellow central area of the retina) and causes it to thicken and swell.

Rose Bengal staining – Staining of the eye to determine if there is adequate mucus and tear film. The test results is a grade in pink color (0-3).

Shirmer's test – Test that determines whether the eye produces enough tears to keep it moist. The result of the test is recorded in terms of # mm of moisture after 5 minutes. (An abnormal result is less than 5 mm of moisture over 5 minutes.)

Tear breakup time (BUT) – there are several types of BUT tests. Basically a measure of time is recorded (in seconds) to determine BUT.

Visual acuity (VA) – VA is acuteness or clearness of vision, which is dependent on the sharpness of the retinal focus within the eye and the sensitivity of the interpretative faculty of the brain. In the expression, 20/20 vision, the numerator refers to the distance in feet between the subject and the chart. The denominator indicates the size of the letters, expressed in millimeters.

Visual field defect – A patient undergoes visual field testing with a Humphrey Field Analyzer (HFA), which uses automated perimetry to measure responses to visual stimuli appearing in central and side vision.

Severity of AMD (Staging) – a gradient scale depicting the severity of AMD. The grades or stages range from 1-5.
