# Supplementary figures and images for: Prototype Development: Context-Driven Dynamic XML Ophthalmologic Data Capture Application
Source: JMIR Med Inform. 2017 Sep 13;5(3):e27. doi: 10.2196/medinform.7465 (PMC5617903; doi:10.2196/medinform.7465)

Database schema used to support the XML-driven data capture framework

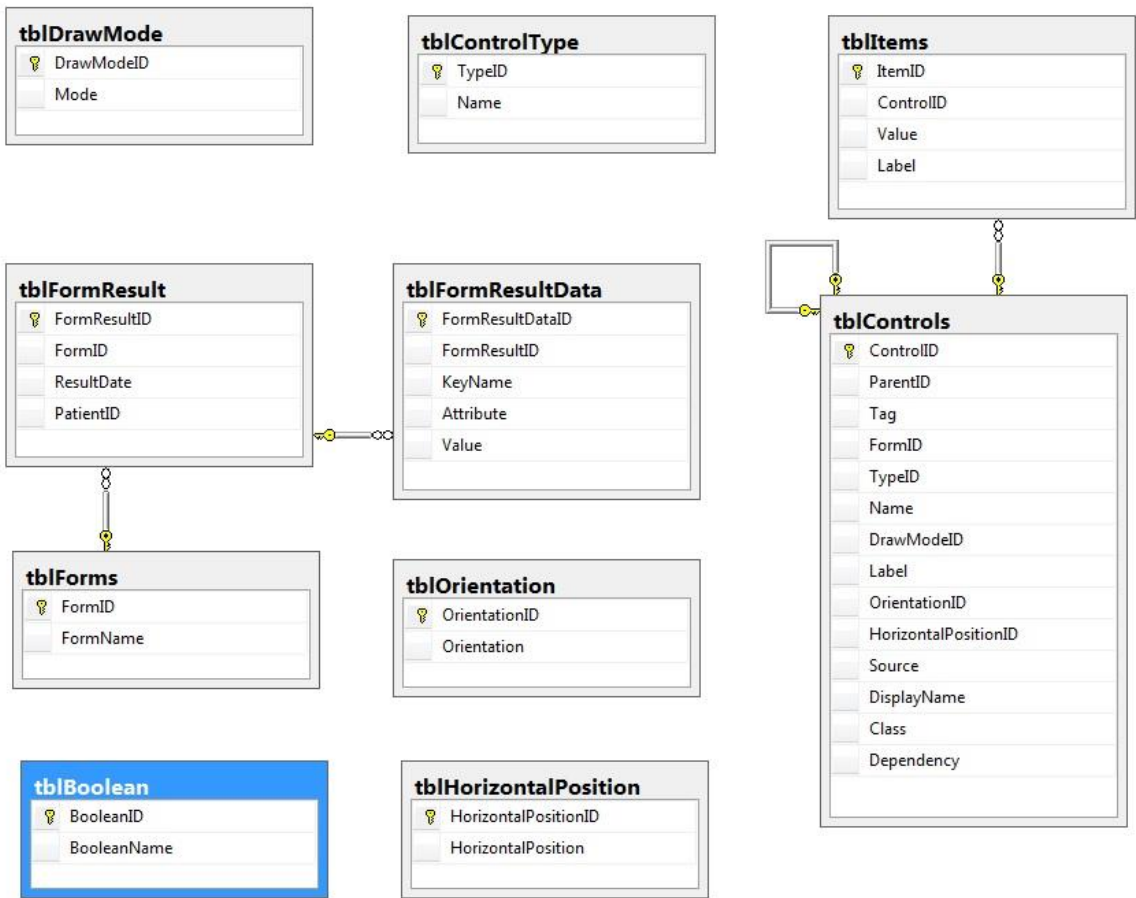

Supplement: Multimedia Appendix 2 [file medinform_v5i3e27_app2.pdf]
